# The effect of regions-of-interest and elasticity modulus selection on differentiating benign and malignant cervical lymph nodes with shear wave elastography

**DOI:** 10.6061/clinics/2020/e1691

**Published:** 2020-10-21

**Authors:** Yong-Mei Sun, Hai Dong, Zong-Yan Du, Zong-Li Yang, Cheng Zhao, Jing Chong, Ping Li

**Affiliations:** I Department of Abdominal Ultrasound, the Affiliated Hospital of Qingdao University, No.16 Jiang Su Rd, Qingdao 266000, Shandong, China; II Department of Intensive Care Unit, the Affiliated Hospital of Qingdao University, No.16 Jiang Su Rd, Qingdao 266000, Shandong, China; III Department of Ultrasound, Maternity and Child Health Care of Zaozhuang, Xuecheng, Zaozhuang 277100, Shandong, China

**Keywords:** Imaging Diagnosis, ROIs, Elasticity Modulus Selection, Shear Wave Elastography

## Abstract

**OBJECTIVE:**

Imaging diagnosis of cervical lymphadenopathy has conventionally used ultrasonography. Shear wave elastography (SWE) is a recent ultrasound technological advancement that has shown promise in the important medical problem of differentiating between benign and malignant cervical lymph nodes based on quantitative measurements of elasticity modulus. However, widely varying elasticity modulus metrics and regions-of-interest (ROIs) were used in existing studies, leading to inconsistent findings and results that are hard to compare with each other.

**METHODS:**

Using a large dataset of 264 cervical lymph nodes from 200 patients, we designed a study comparing three elasticity modulus metrics (Emax, Emean, and standard deviation-SD) with three different ROIs to evaluate the effect of such selections. The metric values were compared between the benign and malignant node groups. The different ROI and metric selections were also compared through receiver operating characteristics curve analysis.

**RESULTS:**

For all ROIs, all metric values were significantly different between the two groups, indicting their diagnostic potential. This was confirmed by the ≥0.80 area under the curve (AUC) values achieved with these metrics. Different ROIs had no effect on Emax, whereas all ROIs achieved high performance at 0.88 AUC. For Emean, the smallest ROI focusing on the area of the highest elasticity achieved the best diagnostic performance. In contrast, the larger ROIs achieved higher performances for SD.

**CONCLUSIONS:**

This study illustrated the effect of elasticity modulus and ROI selection on the diagnostic performance of SWE on cervical lymphadenopathy. These new findings help guide relevant future studies and clinical applications of this important quantitative imaging modality.

## INTRODUCTION

Cervical lymphadenopathy is a common clinical finding, and can result from various conditions such as infection, inflammation, and malignancy (1). Differential diagnosis among these conditions is essential for deciding the subsequent medical management. Differentiating between benign and malignant cervical lymph nodes (LNs) is especially important. Due to its real-time evaluation capability, convenience, and effectiveness, Ultrasonography (US) has conventionally been used as the most important imaging modality for evaluating cervical lymphadenopathy (2). Elastography, a newer US technology that exploits the differences in the elastic properties of soft tissues, has proven useful in this setting (3). Shear wave elastography (SWE) has become especially popular; it displays differences that vary by the propagation through hard or soft tissues after deformation by applying pressure with the ultrasound transducer. By providing quantitative measurements of tissue elasticity (stiffness), SWE has been found useful for imaging diagnosis of many different disease sites such as breast, liver, thyroid, musculoskeletal structures, prostate, as well as cervical LNs (4-18).

The objective of this study is to quantitatively investigate the effects of the varying elasticity modulus metrics and regions-of-interest (ROIs) on differentiating malignant *vs.* benign cervical LNs using SWE. To our knowledge, this is the first such study. Our study was designed using 264 LNs from 200 patients to systematically investigate these effects. The findings can shed light on result comparisons among different studies in which varying elasticity modulus metrics or different ROIs were used and can guide future SWE investigation designs.

## MATERIAL AND METHODS

### General information

A total of 200 patients who were consecutively treated at the University of Qingdao Hospital between June 2017 and March 2019 and met the following inclusion and exclusion criteria were retrospectively studied. Inclusion criteria included enlarged atypical cervical LN on B-mode US, long to short axis ratio <2, presence of echogenic hilus or other ancillary features, and having both B-mode US and SWE examinations with complete cytopathological or histopathological results. Exclusion criteria included LN <0.5 cm from carotid arteries; cystic or largely calcified lesions; no conclusive result of either benign or malignant lymphadenopathies on cytopathological or histopathological examinations; substantial image artifacts or poor image quality on SWE; and previous neck surgery, radiotherapy, or chemotherapy history. Of the 200 patients, 142 are female and 58 are male, with a median (range) age of 45 (14-84). This study was approved by the Institutional Ethics Board and all patients (or guardians) signed the informed consent. In patients with multi-focal lymphadenopathy, only the largest and most visible node for each type was included in the study. This yielded a total of 264 nodes from 200 patients, with a median (range) long axis of 1.5 (0.4-3.1) cm and a median (range) short axis of 0.9 (0.2-1.7) cm.

### Equipment and methods

**Figure 1:**
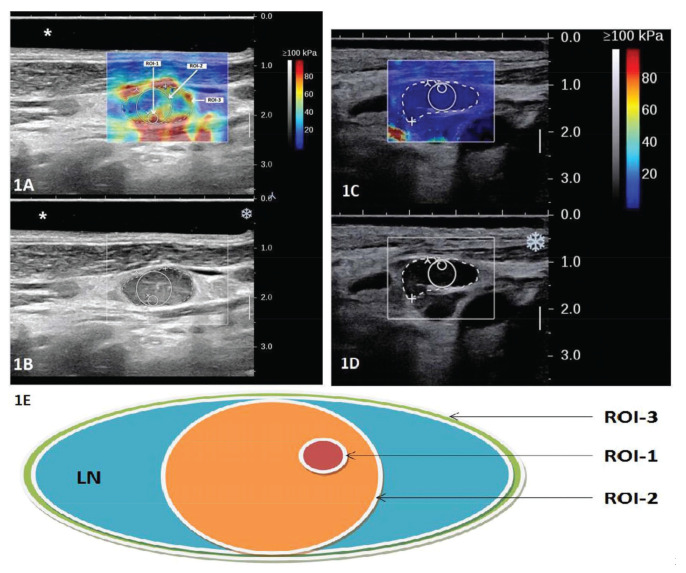
Illustration of the three designed ROIs on overlaid gray-scale and SWE US image (A and C); and gray-scale US image of two example cases (B and D). In the example, the LN shown in A and B is a malignant node from papillary thyroid cancer and the LN shown in C and D is a benign node from reactive hyperplasia. A schematic drawing is shown in E to illustrate the definition of the three ROIs: ROI-1, a small circular ROI with a diameter of 2 mm placed around the stiffest region of the LN; ROI-2, a larger circular ROI tangential to the LN border (with a diameter equal to the thickness of the cortex of the target node) and containing the stiffest region; and ROI-3, a manually drawn ROI encompassing the entire LN.

The patients were imaged with a SuperSonic Aixplorer US system (SuperSonic Imagine, France) using a linear array probe at 4-15 MHz with the preset thyroid protocol. For the exam, patients laid in the supine position with their neck extended to favor acquisition and measurement. A neck cushion was used to assist the gentle neck hyperextension for improved reproducibility. An US gel pad was placed above the imaged LN when necessary. The optimal acoustic window was found, assessed on grayscale imaging, before engaging the SWE™ Mode. The most stable static elastic image was selected, and excess pressure was avoided with appropriate patient positioning and probe holding. The quantitative measurement tool Q-Box^TM^ was employed for data acquisition. Three different ROIs were defined for two example cases as illustrated in Figure 1, in which 1A shows the SWE in color overlay and 1B shows the B-mode US only for a malignant LN from papillary thyroid cancer: ROI-1, a small circular ROI with a diameter of 2 mm placed around the stiffest region of the LN; ROI-2, a larger circular ROI tangential to the LN border (with a diameter equal to the thickness of the cortex of the target node) and containing the stiffest region; and ROI-3, a manually drawn ROI encompassing the entire LN. Similarly, the US/SWE overlay and B-mode US image are shown in Figure 1C and 1D with the three ROIs delineated for a benign LN from reactive hyperplasia. Figure 1E shows a schematic drawing of the 3 ROIs. For each ROI, three repeat measurements were acquired and the average value from the three measurements was recorded for the maximum (Emax), mean (Emean) and standard deviation (SD) of the elasticity. The values and images were collected for subsequent analyses. The operations were jointly performed by two experienced US radiologists in consensus, excluding images that they disagreed on. All LNs included in this study were separately marked and archived after biopsy or resection. When necessary, the US radiologists assisted in the node acquisition. The correspondence between imaging and pathology was ensured via US-guided biopsies for biopsied LNs and careful localization on pre-operative US for surgically removed LNs. Pathological results were obtained from surgical resection when performed, or from biopsy. In most benign cases, the LNs were not surgically removed, but the cytopathological accuracy was ensured with regular patient follow-up examinations.

### Statistical analysis

The normality of the measurands was first assessed with the Shapiro-Wilk test. If the distribution was normal, the measurands were described using χbar±*s*; otherwise, they were described using median and interquartile range (IQR). When comparing non-normally distributed groups, the Mann-Whitney U test was used between two groups while the Kruskal-Wallis H test was used for multiple groups. The histopathological result was used as the gold standard to construct the receiver operating characteristic (ROC) curve for each measurand of each ROI to predict the malignancy of the cervical lymphadenopathy. The area under the ROC curve (AUC) was calculated, and the above tests were performed using SPSS 21.0 (IBM, United States). The ROC curves were compared with each other with a Z test using MedCalc 19.1 (MedCalc Software, Belgium). A *p*<*0.05* was considered statistically significant for all tests.

## RESULTS

A total of 264 cervical LNs from 200 patients were included in the study. Table 1 describes the cytopathology/histopathology of the included LNs, of which 161 were malignant (61%) and 103 were benign (39%). A large portion of the malignant LNs were from papillary thyroid cancer. The patient distribution of the LNs is also reported in Table 1; 116 had malignant LNs only (58%), 77 had benign LNs only (38.5%), and seven had both malignant and benign LNs (3.5%).

### Elasticity modulus comparison between benign and malignant LNs

The median and interquartile range (IQR) values of all elasticity modulus metrics and their comparisons are reported in Table 2 for benign and malignant LNs. For all metrics from all ROIs, statistically significant differences were observed between the benign and malignant LNs, confirming the usefulness of SWE in quantitatively differentiating the two groups. Regardless of the selected ROI, Emean, Emax, and SD were all higher in the malignant group than in the benign group. Using ROI-1 as an example, the median Emax was 51.3 kPa and 22.1 kPa for malignant and benign LNs, respectively; the median Emean was 44.3 *vs.* 17.3 kPa; and the median SD was 4.8 kPa *vs.* 2.1 kPa. The higher Emean and Emax suggest that the malignant LNs tend to be stiffer than the benign LNs, and the higher SD indicates a higher intralesional heterogeneity in malignant LNs.

### Elasticity modulus comparison between different ROIs

The ROI comparison results are also shown in Table 2. For Emax values, no significant difference was observed among the three ROIs, with a median value of 39.4 kPa, 39.4 kPa, and 40.8 kPa for ROI-1, ROI-2, and ROI-3, respectively. For Emean and SD, the differences were significant for all LNs as well as for benign and malignant LNs. ROI-1, the smallest ROI of the three, showed the highest Emean among the three ROIs (with a median value over all LNs of 27.1 kPa compared with ROI-2 at 20.7 kPa and ROI-3 at 18.7 kPa) and the smallest SD (with a median value over all LNs of 3.2 kPa compared with ROI-2 at 5.7 kPa and ROI-3 at 6.3 kPa). The same observations were also found within the benign group as well as within the malignant group, although the variation was larger in the malignant group than that in the benign group. For example, the median Emean values were 44.3 kPa, 31.2 kPa, and 28.1 kPa for malignant LNs in ROI-1, 2, and 3, respectively; compared with 17.3 kPa, 15.0 kPa, and 13.9 kPa for benign LNs. Notably, ROI-3, the largest ROI encompassing the entire LN, showed the largest SD among the three ROIs for the malignant group (at a median value of 9.0 kPa *vs.* the other two ROIs at 4.8 and 7.6 kPa), but with smaller differences for the benign group (at a median value of 3.1 kPa *vs.* the other two ROIs at 2.1 and 2.8 kPa). This agrees with the benign *vs.* malignant group comparison that showed that benign LNs are more homogeneous.

### Diagnostic performance of each elasticity modulus and comparison between ROIs

**Figure 2:**
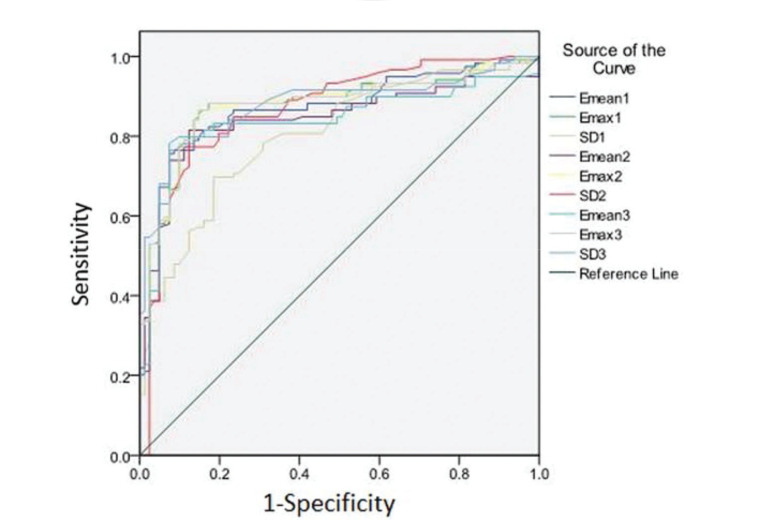
Comparison of the diagnostic performances in predicting malignant LNs among the three elasticity modulus metrics from the three ROIs.

The performance of SWE for differentiating benign *vs.* malignant groups using Emax, Emean, and SD was good. The ROC curves are plotted in Figure 2 and the AUC results are listed in Table 3. From the ROC analysis, they all achieved AUC values higher than or equal to 0.8. When comparing different ROIs, Emax showed no statistical difference between the three ROIs, all achieving the highest AUC among the three elasticity modulus metrics at around 0.88. For Emean, ROI-1 performed significantly better than the other two ROIs at an AUC of 0.87. In contrast for SD, ROI-1 performed significantly worse than the other two ROIs at an AUC of 0.80.

## DISCUSSION

SWE is a recent US technological advancement, the diagnostic value of which has been found for several diseases including cervical lymphadenopathy. A rapidly increasing studies have been reported on using SWE to diagnose cervical lymphadenopathy (13-22). Many recent studies have demonstrated its usefulness in discriminating malignant from benign cervical LNs (13,16,18,20,22,24). However, although most of these studies demonstrated the ability of SWE in differentiating malignant cervical LNs from benign ones as well as in even more challenging subgroups, a standard quantitative elasticity modulus metric and a recommended threshold have not been established, especially in terms of the quantitative metric used for measuring elasticity modulus and selected ROI. Furthermore, inconsistent performances of SWE were also reported (13,14,20). A few studies reported an improvement of diagnostic accuracy with SWE compared with conventional US (13,20), while another study failed to show that (14). Using 67 LNs, Choi et al. reported the superior performance of SWE compared with B-mode US in terms of accuracy, sensitivity, and specificity for differentiating malignant *vs.* benign cervical LNs (20,25). Desmots et al. reported a similar finding in another study on 62 LNs (13). However, Kang et al. reported the opposite finding in a study of 130 LNs (14). Biological heterogeneity in the samples from different studies could partially contribute to the observed differences, such as the cell type and histology of the studied LNs. At the same time, the variance in the selection of ROI and elasticity modulus metric could also play a role. ROI size was found to influence SWE’s diagnostic performance on breast cancer in a few recent studies (7,23). But to the best of our knowledge, no study on the effect of this for cervical LN applications has been reported. In fact, a meta-analysis by Suh et al. suggested that ROI selection may be one of the contributing causes for the diagnostic performance variability of SWE on cervical LNs (24). Therefore, an investigation on the influence of ROI and elasticity modulus metric selection is important and needed for diagnosing cervical LNs with SWE. To our knowledge, our large-cohort study performed on 264 cervical LNs of 200 patients is the first reported effort on this meaningful investigation.

ROIs of varying sizes have been used in previous studies. Some used small circular ROIs with a diameter of 1-3 mm (21), while others used bigger ROIs up to those that encompass the whole LN (13,18,20,25). In our study, we compared ROIs of three different sizes including two circular ROIs of varying diameters and one ROI delineating the entire LN. Similarly, while many studies used the maximum shear elasticity modulus (Emax) inside the ROI (13,16,20,21), other elasticity modulus metrics such as the mean (Emean) and SD of the ROI were also used (16-18,22). For each metric, varying cutoff values were recommended by different studies. Therefore, we also investigated different elasticity modulus metrics including Emax, Emean, and SD. Our results showed that there were significant differences between the benign and malignant LNs for all metrics from all ROIs, and malignant LNs were stiffer and more heterogeneous than their benign counterparts. This was confirmed by the good performances (≥0.80 AUC) of all metrics in the ROC analysis to differentiate the two groups of LNs. It can also be visually observed on the example elastogram of a papillary thyroid cancer malignant LN in Figure 1A and that of a reactive hyperplasia benign LN in Figure 1C, where the malignant LN is visibly much stiffer (with higher elasticity values, indicated by warmer colors in Figure 1A, in some regions) and heterogeneous than the benign LN. Among them, Emax was similar among different ROIs, which is expected as the smaller ROIs were designed to focus on the stiffest region. Comparing different elasticity modulus metrics, Emax also achieved the highest AUCs at 0.88, suggesting that Emax would be a good SWE metric to use for diagnosing cervical LNs, and its value is fairly robust against different ROI selection so inter-study comparison of performances and cutoff values with this metric would be most feasible. For Emean, ROI-1 (the smallest ROI with a 2 mm-diameter circle) achieved a significantly higher AUC at 0.87 than the other two ROIs. This also appears to be consistent with the established observations that malignant LNs are stiffer than benign ones and are more heterogeneous due to factors such as microcalcification, necrosis, and cystic changes (2,15). Therefore, when using Emean as the diagnostic metric, smaller ROI focused on areas of the highest stiffness is recommended. SD has also been used as an evaluation metric by previous studies (15,17,18). In our study, we found that SD performed better with the larger ROIs in the differential diagnosis task.

Our study results meaningfully illustrated the effect of elasticity modulus selection and ROI selection. They are helpful not only for cross-comparing existing studies, but also for designing a future study. However, it is also important to note that in addition to these two factors, there are also other variables not addressed by our study that could affect the quantitative application of SWE for cervical lymphadenopathy. For example, there could be inherent differences coming from the histopathology of the LNs. Furthermore, artifacts could also arise that affect the quantitative accuracy of SWE due to the non-uniform probe pressure from uneven cervical anatomy. Lastly, our study was not designed to address the possible effects of microcalcification, necrosis, and cystic changes in the LN, although an effort was made in our study to exclude these from the selected ROIs.

## CONCLUSIONS

The selection of different ROIs and elasticity modulus metrics affect the performance results of SWE in cervical lymph node diagnosis. The max, mean, and SD of the elasticity modulus are all good SWE metrics for differentiating benign and malignant nodes. Of them, the max value yielded the best performance in our cohort and is independent of ROI selection, achieving AUCs of 0.88 regardless of ROI selection in our cohort and with median values of about 51.3 kPa and 22.1 kPa for malignant *vs.* benign LNs, respectively. Small ROIs are more suitable when using the mean value, achieving the highest AUC of 0.87 with the smallest ROI at median values of 44.3 kPa and 17.3 kPa for malignant *vs.* benign LNs, respectively; and larger ROIs are better when using the SD, achieving the highest AUC of 0.88 with the largest ROI at median values of 9.0 kPa and 3.1 kPa for malignant *vs.* benign LNs, respectively.

## AUTHOR CONTRIBUTIONS

Sun YM and Dong H, contributed to primary researchers, writers, researched and wrote significant portions of introduction, editing, and revising. Du ZY and Yang ZL, wrote a section on technical aspects of reduction which was not included; participated in revision process. Zhao C, Li P, and Chong J, provided necessary oversight and guidance and focused the work on the most relevant references, concepts, and controversies; heavily involved in editing and revision.

## Figures and Tables

**Table 1:** Cytopathology/Histopathology of the 264 cervical LNs included in the study and the patient distribution of the LNs.

	Cytopathology/Histopathology	Number	%
Malignant LNs		161	61
	Papillary Thyroid Cancer (PTC)	109	41
	Nasopharyngeal Squamous Cell Carcinoma	7	3
	Lymphoma	10	4
	Oropharyngeal Squamous Cell Carcinoma	4	2
	Squamous Cell Lung Cancer	6	2
	Lung Adenocarcinoma	22	8
	Pancreatic Cancer	1	0
	Undifferentiated Thyroid Cancer	2	1
Benign LNs		103	39
	Reactive Hyperplasia	89	34
	Tuberculous Lymphadenitis	14	5
Patients		200	100
	Malignant LNs Only	116	58
	Benign LNs Only	77	38.5
	Malignant and Benign LNs	7	3.5

**Table 2:** Shear elasticity modulus values (unit: kPa) from different ROIs and for benign vs. malignant LNs. The values are all described in median (interquartile range). The comparison statistics between benign and malignant LNs are listed to the right of the columns, and those among the three ROIs are listed in the rows below each elasticity modulus metric.

Elasticity Modulus Metric	All LNs	Benign LNs	Malignant LNs	Z Value	*P* Value
Emax					
1	39.40 (22.10-65.83)	22.10 (18.50-26.20)	51.30 (39.00-82.80)	9.090	0.000
2	39.35 (22.25-66.00)	22.10 (19.25-26.50)	51.30 (38.90-82.80)	9.067	0.000
3	40.80 (22.05-66.40)	22.10 (19.45-26.60)	51.70 (38.90-82.80)	9.036	0.000
H Value	0.04	0.148	0.026		
*P* Value	0.98	0.929	0.987		
Emean					
1	27.05 (16.98-50.35)	17.30 (13.60-21.20)	44.30 (26.40-62.10)	8.837	0.000
2	20.70 (14.20-39.18)	15.00 (11.50-18.95)	31.20 (20.70-44.30)	8.205	0.000
3	18.70 (12.50-32.40)	13.90 (10.10-15.80)	28.10 (18.60-37.80)	8.852	0.000
H Value	28.71	24.4	34.17		
*P* Value	0.000	0.000	0.000		
SD					
1	3.20 (1.80-6.80)	2.10 (1.25-3.05)	4.80 (2.80-9.70)	7.250	0.000
2	5.70 (2.80-9.30)	2.80 (1.80-4.10)	7.60 (5.50-12.00)	8.865	0.000
3	6.25 (3.10-10.10)	3.10 (2.40-3.90)	9.00 (6.20-12.70)	9.070	0.000
H Value	32.66	22.44	26.27		
*P* Value	0.000	0.000	0.000		

**Table 3:** Diagnostic performance of the shear elasticity modulus metrics from each ROI in differentiating malignant from benign LNs.

Elasticity Modulus Metric	Cutoff Value (kPa)	AUC	95%CI	Paired Comparison	Z Value	*P* Value
Emax1	29.20	0.879	0.826-0.921	Emax1 vs Emax2	0.593	0.5531
Emax2	31.95	0.878	0.824-0.920	Emax1 vs Emax3	1.825	0.6801
Emax3	31.15	0.877	0.823-0.919	Emax2 vs Emax3	0.742	0.4582
Emean1	24.40	0.868	0.813-0.912	Emean1 vs Emean2	2.176	0.0296
Emean2	20.60	0.842	0.784-0.890	Emean1 vs Emean3	2.296	0.0217
Emean3	20.45	0.839	0.781-0.887	Emean2 vs Emean3	0.611	0.5411
SD1	3.35	0.802	0.740-0.855	SD 1 vs SD 2	2.751	0.0059
SD2	5.35	0.869	0.815-0.913	SD 1 vs SD 3	2.744	0.0061
SD3	6.40	0.878	0.824-0.920	SD 2 vs SD 3	0.436	0.6629
